# Characterization of Macrophage-Like Cells in Retinal Vein Occlusion Using En Face Optical Coherence Tomography

**DOI:** 10.3389/fimmu.2022.855466

**Published:** 2022-03-03

**Authors:** Yunkao Zeng, Xiongze Zhang, Lan Mi, Yuhong Gan, Yongyue Su, Miaoling Li, Ruijun Yang, Yining Zhang, Feng Wen

**Affiliations:** State Key Laboratory of Ophthalmology, Zhongshan Ophthalmic Center, Sun Yat-sen University, Guangdong Provincial Key Laboratory of Ophthalmology and Visual Science, Guangdong Provincial Clinical Research Center for Ocular Diseases, Guangzhou, China

**Keywords:** retinal vein occlusion, microglia, inflammation, optical coherence tomography, macrophage-like cells

## Abstract

**Purpose:**

To investigate the clinical features of a macrophage-like cell (MLC) obtained by en face optical coherence tomography (OCT) in retinal vein occlusion (RVO).

**Methods:**

The study involved 36 patients with treatment-naïve unilateral acute RVO, including 21 branch RVO (BRVO) and 15 central RVO. Vessel density and macular thickness were quantified using OCT angiography. A 3-μm en face OCT slab on the inner limiting membrane in the optic nerve head (ONH) region or macular region was used to visualize the MLCs. The MLCs were binarized and quantified using a semiautomated method. The unaffected fellow eyes served as the control group.

**Results:**

The morphology of MLCs appeared larger and plumper in RVO eyes. The mean MLC density in the ONH and macular regions was 2.46 times and 2.86 times higher than their fellow eyes, respectively (*p* < 0.001). The macular MLC density of the occlusive region was significantly lower than that of the unaffected region in BRVO (*p* = 0.01). The ONH and macular MLC densities in the non-perfused region were significantly lower than those in the perfused region in all RVO eyes (*p* < 0.001). The ONH MLC density in RVO eyes was negatively correlated with radial peripapillary capillary vessel density (r = −0.413, *p* = 0.012). Both ONH and macular MLC densities were positively correlated with macular thickness (r = 0.505, *p* = 0.002; r = 0.385, *p* = 0.02, respectively).

**Conclusion:**

The increased density and changes of morphology characterized by OCT may indicate generalized activation and aggregation of MLCs in RVO. More MLCs are recruited in the perfused region rather than the non-perfused region. RVO eyes with a higher density of MLCs tend to suffer from the thicker macula.

## Introduction

Retinal vein occlusion (RVO) is the second most common retinal vascular disease after diabetic retinopathy, and it is an important cause of visual impairment (1). RVO can be classified as branch RVO (BRVO) and central RVO (CRVO). It was estimated that in 2015, the global prevalence of any RVO in a population aged 30–89 years was 0.77%, which was equivalent to an overall of 28.06 million people (2). A previous study reported that RVO leads to retinal tissue ischemia and hypoxia, followed by inflammation related to macrophage-like cells (MLCs) like microglia and hyalocytes (3). Hyalocytes are considered resident macrophages of cortical vitreous, which can be an exacerbating factor in eyes with inflammation (4). Under normal situations, hyalocytes are mainly distributed close to the inner limiting membrane in the posterior hyaloid and at the vitreous base (4). Previous studies found that hyalocytes play important roles in various aspects of pathophysiology, which involve diabetic macular edema, epiretinal membrane formation, proliferative vitreoretinopathy, and proliferative diabetic retinopathy (4–8). Microglia is a type of macrophage located primarily in the inner plexiform layer and outer plexiform layer of the normal retina. A smaller proportion also resides in the ganglion cell layer, nerve fiber layer, and near the inner limiting membrane (9, 10). Under pathologic situations, the microglia is able to proliferate and move rapidly towards damaged areas and resolve tissue damage (11).

The role of MLCs in the clinical consequences of retinal diseases is a topic of growing interest. Despite the essential roles they played in retinal diseases, labeling was required to visualize MLCs, and thus they were studied predominantly *ex vivo* or in animal models (10). The advancement of label-free adaptive optics optical coherence tomography (OCT) allows us to visualize human retinal macrophage near the inner limiting membrane (10). However, the cost and technical complexity limit the availability of adaptive optics imaging for clinical applications. In terms of clinical OCT, previous studies argued that intraretinal hyperreflective dots on the B-scan of OCT might represent inflammatory cells like macrophage and activated microglia in patients with RVO and diabetic retinopathy (12, 13). However, the origin of intraretinal hyperreflective dots is still being debated, for they may also represent lipid extravasation rather than activated microglia (12, 14).

Recently, Castanos et al. presented a novel *in vivo* imaging method to visualize individual MLCs on the inner limiting membrane of the human retina using clinical en face OCT (15). Detecting the MLCs on the inner limiting membrane may also avoid the influence of lipid exudation and thus ensure the accuracy of detecting MLCs. The *in vivo* research on the characteristics of MLCs is limited in human RVO. The current study aimed to visualize and characterize MLCs on the inner limiting membrane of RVO eyes using en face OCT.

## Materials and Methods

### Subjects

This was a cross-sectional study of treatment-naïve patients with unilateral RVO included between June 2021 and October 2021 at Zhongshan Ophthalmic Center, China. Patients with a disease duration of less than 2 months were included in the study. The current study was approved by the Institutional Review Board of the Zhongshan Ophthalmic Center and adhered to the tenets of the Declaration of Helsinki. All subjects underwent complete ocular examination, including intraocular pressure, refractive error (autorefractometry), best-corrected visual acuity, slit-lamp fundus examination, fundus photography, and OCT angiography (OCTA). Excluded criteria were as follows: 1) subjects with refractive error >3 diopters [D]; 2) subjects with any other ocular conditions affecting the neural and vascular structures of the eye (glaucoma, uveitis, diabetic retinopathy, etc.); 3) history of ocular trauma, ocular surgery, laser photocoagulation, anti-vascular endothelial growth factor (anti-VEGF), or steroid treatment; and 4) OCTA images with poor quality (scan quality <6 or obvious motion artifact).

### Optical Coherence Tomography Angiography Imaging and Processing

The optic nerve head (ONH) and macular parameters were obtained with the AngioVue OCTA system (RTVue-XR Avanti; Optovue, Fremont, CA, USA, Version 2017.1.0.151). High-density (HD) macular 6 × 6 mm program was chosen to measure averaged macular thickness and macular vessel density in both the superficial capillary plexus (SCP) and deep capillary plexus (DCP). HD disc 4.5 × 4.5 mm program was used to obtain radial peripapillary capillary vessel density. To achieve a better imaging quality, two orthogonal OCTA volumes were obtained at the same location and then registered using motion correction technology (16). After image acquisition, OCTA images and en face OCT images were generated. The vessel densities in the ONH and macular regions were automatically calculated by AngioVue OCTA. The value of macular thickness in the 6 × 6 mm macular region was also generated by the device. Radial peripapillary capillary vessel density in the whole 4.5 × 4.5 mm region and macular vessel density and macular thickness in the whole 6 × 6 mm region were used in the subsequent analysis. The segmentation of the retina was checked and corrected manually if they deviated from the right position.

In order to visualize the MLCs on the inner limiting membrane, a 3-μm slab above the inner limiting membrane on the ONH and macular regions was manually adjusted as previously described (15). This specific layer is more likely to capture MLCs on en face OCT since better structural contrast can be achieved. In order to cover a larger retinal area, we chose the HD 6 × 6 mm image instead of the ordinary 3 × 3 mm image. The retinal nerve fiber layer located from 0 to 28 μm below the inner limiting membrane was also segmented. The MLCs on the extracted 3-μm slab were identified and isolated using a semiautomated binarization process with ImageJ as previously described in a masked fashion (17, 18). Briefly, the extracted images underwent noise reduction to remove background irregularities and vessel artifacts, signal enhancement to improve cell identification, and binarization to extract discrete cell shapes (18). The analyzed particles function of ImageJ was used to calculate the number of MLCs from binarized images. We also measured the area of the non-perfusion region (region without visible capillary), the area of the affected region (the region that the obstructive vein previously supplied), and the unaffected region using ImageJ. The density of MLCs was defined as the number of cells over their corresponding image area.

### Statistical Analysis

Data analyses were performed using SPSS software version 19.0 (SPSS Inc., Chicago, IL, USA). Demographic and outcome data were summarized by frequency for categorical variables and mean ± SD for continuous variables. The Shapiro–Wilk test was used to test the normality of the data. We performed paired t-test for parametric data. The differences of MLC parameters between RVO eyes and their fellow eyes were compared using Wilcoxon signed-rank test. Differences between the perfused and non-perfused regions in all RVO eyes and the affected and unaffected regions in the BRVO eyes were also compared using the Wilcoxon signed-rank test. Pearson’s correlation coefficient was used to evaluate the linear correlation between MLC density, vessel density, and macular thickness. *p* < 0.05 was considered statistically significant.

## Results

Seventy-two eyes of 36 unilateral RVO patients, including 21 BRVO and 15 CRVO, were involved in the study. Their unaffected 36 fellow eyes served as the control group. The mean age of the included patients was 50.72 ± 11.54 years, and women accounted for 38.89% (14/36).

**Figure 1:**
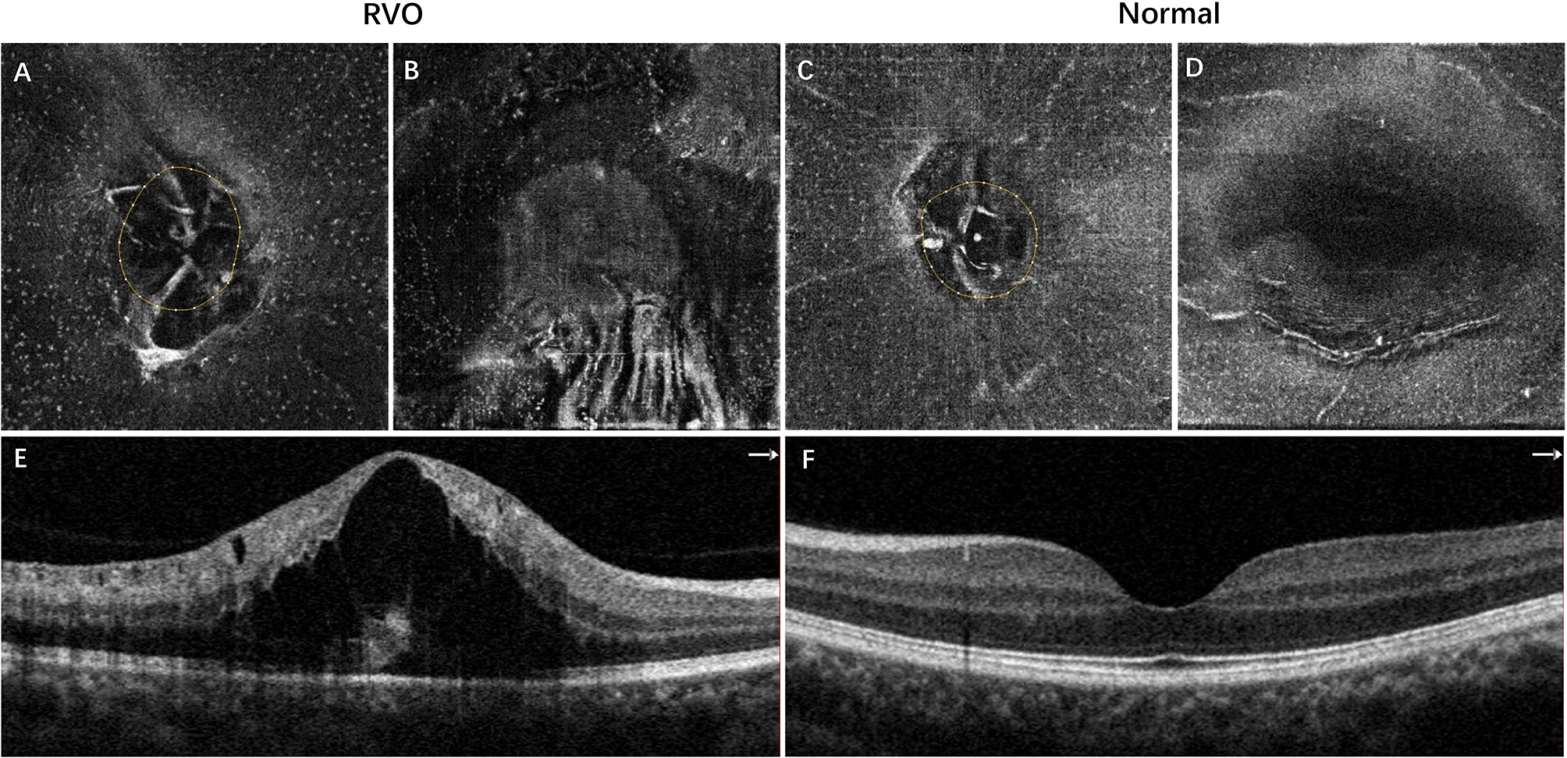
Representative example of inferotemporal BRVO in a right eye **(A, B, E)** and the normal fellow eye **(C, D, F)**. **(A, C)** The 3-μm en face OCT slabs showing MLCs (white dots) in the ONH region. **(B, D)** The 3-μm en face OCT slabs showing MLCs in the macular region. **(E)** OCT B scan showing cystoid macular edema of BRVO. **(F)** Normal OCT B scan of the normal eye. The MLCs were more abundant in both ONH and macular regions in the eye with BRVO. BRVO, branch retinal vein occlusion; OCT, optical coherence tomography; MLCs, macrophage-like cells; ONH, optic nerve head.

**Figure 2:**
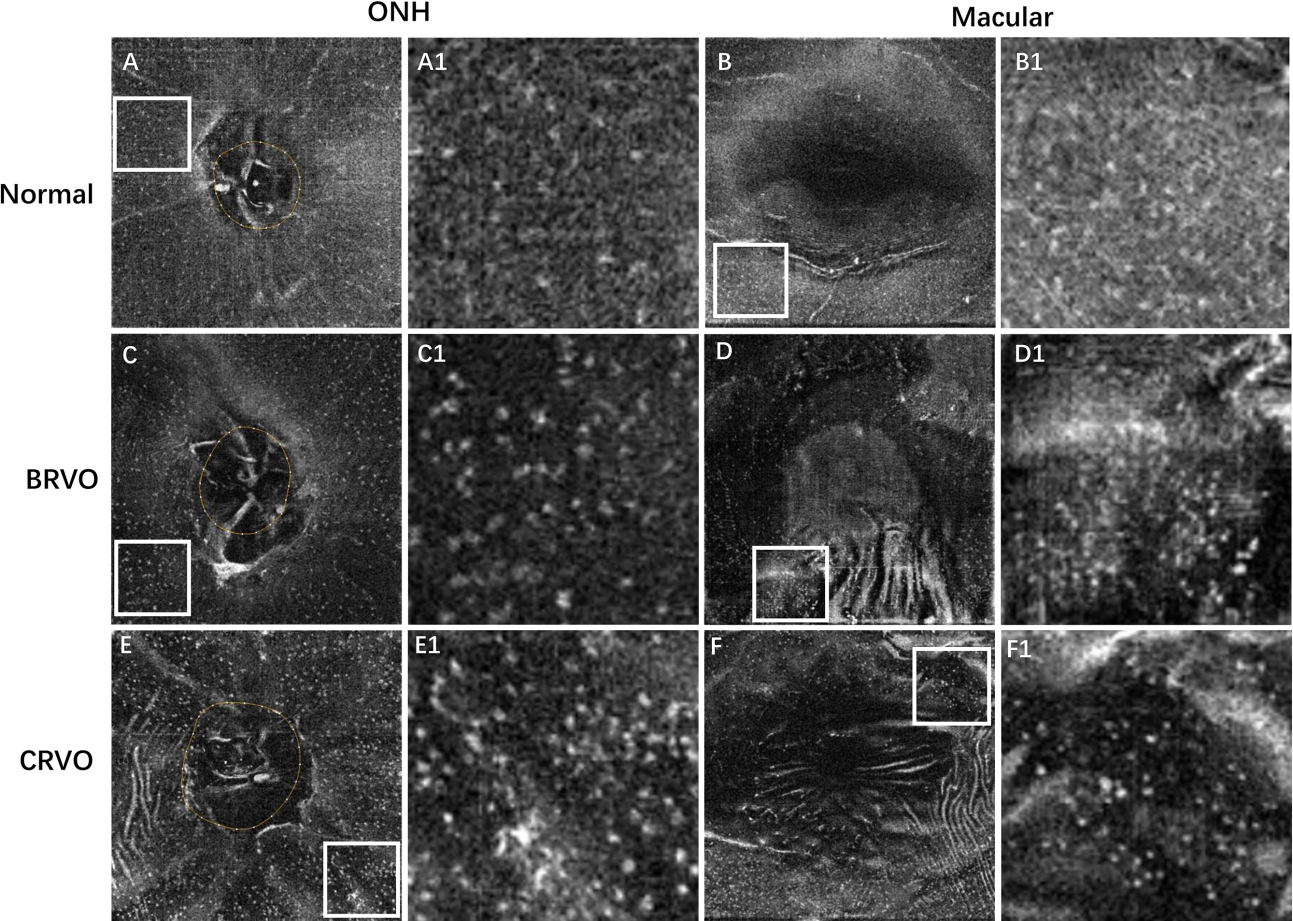
Representative examples showing the cellular morphology in a normal eye (first row), a patient with BRVO (second row), and a patient with CRVO (last row). The first and second columns show the en face OCT of the ONH and macular regions, respectively. The regions of interest (ROI) with relatively higher cell densities in 4.5 × 4.5 mm ONH region and 6 × 6 mm macular region were indicated by white box. The ROI was enlarged by 4 times and shown at the right side of the original figures. The MLCs appear larger and plumper with fewer protrusions in eyes with RVO. BRVO, branch retinal vein occlusion; CRVO, central retinal vein occlusion; OCT, optical coherence tomography; ONH, optic nerve head; MLCs, macrophage-like cells.

The comparisons of MLCs and retinal parameters are shown in **Table 1**. The radial peripapillary capillary vessel density in the whole ONH region was significantly lower in RVO eyes compared with the fellow eyes (45.70 ± 4.12 vs. 49.65 ± 2.93, *p* < 0.001). Similarly, macular vessel densities in both the SCP and DCP were significantly lower in the RVO eyes (45.51 ± 3.46 vs. 48.75 ± 3.74, *p* = 0.002 and 43.62 ± 4.68 vs. 47.32 ± 5.86, *p* = 0.003 respectively). Conversely, the macular thickness of the RVO eyes was significantly higher than that of their fellow eyes (374.50 ± 64.25 vs. 287.42 ± 13.17 μm, *p* < 0.001). The number of ONH and macular MLCs of all RVO eyes were elevated in comparison to the fellow eyes (261.75 ± 119.77 vs. 106.58 ± 64.68, *p* < 0.001 and 459.43 ± 264.78 vs. 160.44 ± 116.41, *p* < 0.001 respectively). The mean ONH MLC density was 2.46 times, while the mean macular MLC density was 2.86 times higher than that of the control group (12.93 ± 5.91 vs. 5.26 ± 3.19 cells/mm^2^, *p* < 0.001 and 12.76 ± 7.36 vs. 4.46 ± 3.23 cells/mm^2^, *p* < 0.001). A representative example is shown in **Figure 1**. Apart from the increased number and density of MLCs, changes in MLC morphology were also observed in RVO. They appeared slender with spindle- or star-like configuration in fellow eyes, while their morphology transformed into a larger and plumper appearance with fewer protrusions in eyes with RVO (**Figure 2**).

**Table 1:** MLC parameters and retinal parameters in RVO eyes and the fellow eyes.

	RVO eyes (n = 36)			Fellow eyes	*p*
	BRVO (n = 21)	CRVO (n = 15)	All RVO eyes	(n = 36)	
RPC VD	47.83 ± 2.63	42.70 ± 4.02	45.70 ± 4.12	49.65 ± 2.93	<0.001^†^
Macular VD in SCP	44.81 ± 2.82	46.49 ± 4.11	45.51 ± 3.46	48.75 ± 3.74	0.002^†^
Macular VD in DCP	44.26 ± 3.97	42.71 ± 5.56	43.62 ± 4.68	47.32 ± 5.86	0.003^†^
Macular thickness (μm)	362.57 ± 60.26	391.20 ± 67.97	374.50 ± 64.25	287.42 ± 13.17	<0.001^†^
ONH MLC count	233.52 ± 114.61	301.27 ± 119.27	261.75 ± 119.77	106.58 ± 64.68	<0.001^‡^
ONH MLC density (cells/mm^2^)	11.53 ± 5.66	14.88 ± 5.89	12.93 ± 5.91	5.26 ± 3.19	<0.001^‡^
Macular MLC count	395.18 ± 250.87	549.38 ± 265.53	459.43 ± 264.78	160.44 ± 116.41	<0.001^‡^
Macular MLC density (cells/mm^2^)	10.98 ± 6.97	15.26 ± 7.38	12.76 ± 7.36	4.46 ± 3.23	<0.001^‡^

MLCs, macrophage-like cells; BRVO, branch retinal vein occlusion; CRVO, central retinal vein occlusion; RPC, radial peripapillary capillary; VD, vessel density; SCP, superficial capillary plexus; DCP, deep capillary plexus; ONH, optic nerve head.

^†^Compared using paired t-test between all RVO eyes and fellow eyes.

^‡^compared using the Wilcoxon signed rank test between all RVO eyes and fellow eyes.

**Figure 3:**
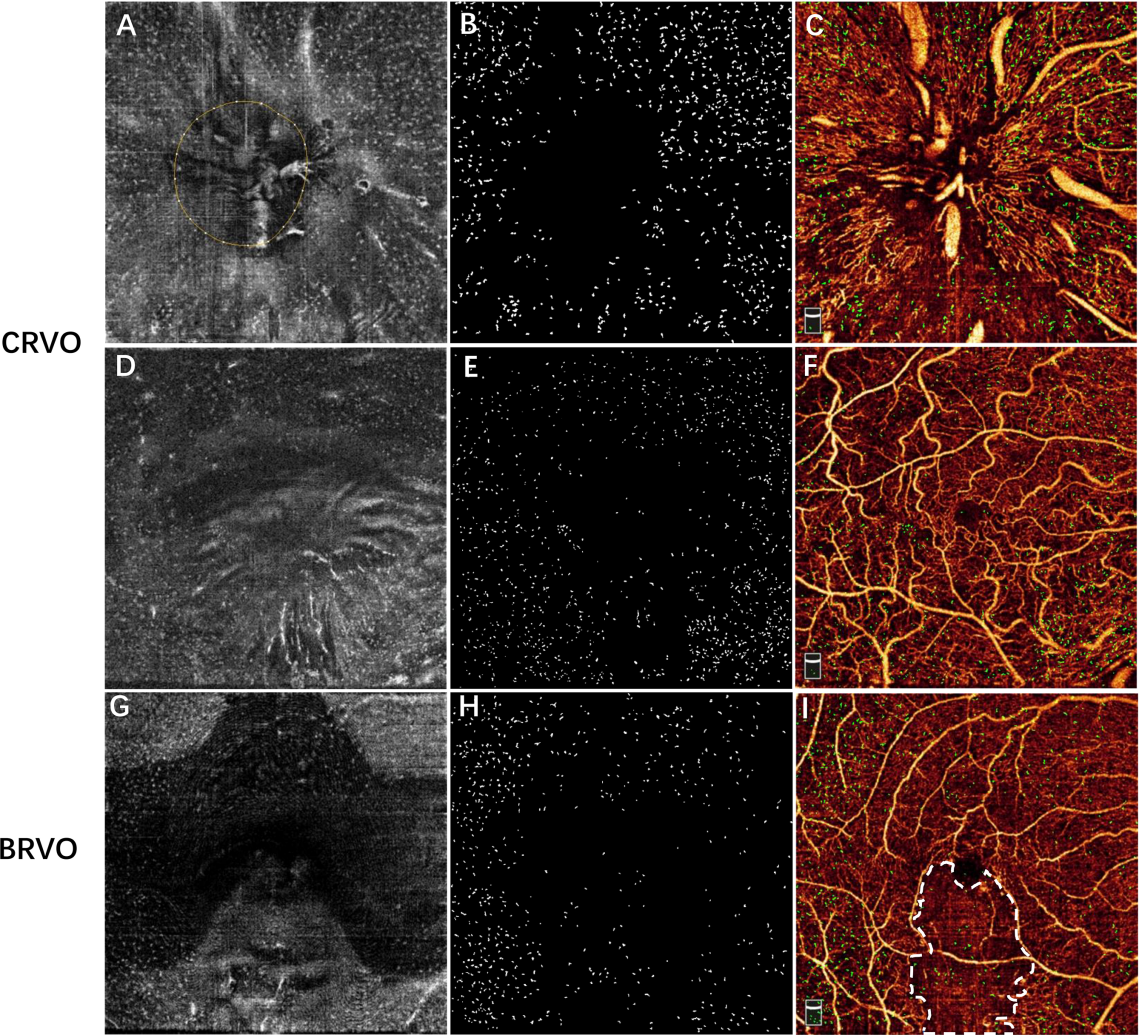
Examples showing the distribution of MLCs relative to the vessels in a patient with CRVO **(A–F)** and a patient with inferotemporal BRVO **(G–I)**. The first row shows the images of the ONH region, and the second and third rows show the images of macular region. **(A, D, G)** The 3-μm en face OCT slab showing macrophage-like cells. **(B, E, H)** Subtracted and enhanced MLCs. **(C, F, I)** Overlay of vascular network and macrophage-like cells (green). **(I)** En face OCTA image showing the non-perfused region (region inside white dash). Note that MLCs were sparse in the central macular and non-perfused regions of RVO eye. MLCs, macrophage-like cells; CRVO, central retinal vein occlusion; BRVO, branch retinal vein occlusion; ONH, optic nerve head; OCT, optical coherence tomography; OCTA, optical coherence tomography.

With regard to the distribution of MLCs in the perfused and non-perfused regions, the cellular measurements and retinal parameters are shown in **Table 2**. We found that 12 eyes exhibited retinal non-perfusion in the ONH region, while 29 eyes exhibited retinal non-perfusion in the macular region among the 36 RVO eyes. Both the ONH and macular MLC densities in the non-perfused region were significantly lower than the perfused region (6.35 ± 4.96 vs. 13.56 ± 5.24 cell/mm^2^, *p* = 0.002 and 5.75 ± 4.56 vs. 13.87 ± 7.93 cell/mm^2^, *p* < 0.001 respectively). An example showing the distribution of MLCs is shown in **Figure 3**. With regard to the distribution of MLCs in the occlusion-affected and unaffected regions, the cellular measurements and retinal parameters are shown in **Table 3**. The MLC densities in the occlusion-affected ONH region and unaffected region were comparable (11.05 ± 7.77 vs. 10.33 ± 4.44 cells/mm^2^, *p* = 0.999). The density of MLCs in the unaffected macular region was significantly higher than that of the affected macular region in BRVO eyes (12.17 ± 8.06 vs. 8.71 ± 6.60 cells/mm^2^, *p* = 0.01). However, the MLC densities of the affected ONH and macular regions were still significantly higher than those of their fellow eyes (11.05 ± 7.77 vs. 3.68 ± 1.75 and 8.71 ± 6.60 vs. 3.77 ± 2.25 cells/mm^2^, both *p* = 0.001).

**Table 2:** MLC parameters in non-perfused and perfused regions of the RVO eyes.

	Non-perfused region	Perfused region	*p*
ONH region (n = 12)			
MLC count	9.92 ± 8.88	247.58 ± 97.87	0.002^†^
Area (mm^2^)	1.87 ± 1.88	18.38 ± 1.88	0.002^†^
MLC density (cells/mm^2^)	6.35 ± 4.96	13.56 ± 5.24	0.002^†^
Macular region (n = 29)			
MLC count	21.31 ± 26.22	450.03 ± 265.70	<0.001^†^
Area (mm^2^)	3.64 ± 3.18	32.46 ± 3.32	<0.001^†^
MLC density (cells/mm^2^)	5.75 ± 4.56	13.87 ± 7.93	<0.001^†^

MLC, macrophage-like cell; RVO, retinal vein occlusion; ONH, optic nerve head.

^†^Compared using Wilcoxon signed-rank test.

**Table 3:** MLC parameters and retinal parameters in the occlusion-affected and unaffected regions of BRVO eyes.

	Affected region	Unaffected region	*p*
ONH region (n = 15)			
MLC count	42.87 ± 53.03	166.27 ± 42.15	0.001^†^
Area (mm^2^)	3.29 ± 2.86	16.96 ± 2.86	0.001^†^
MLC density (cells/mm^2^)	11.05 ± 7.77	10.33 ± 4.44	0.999^†^
Macular region (n = 21)			
MLC count	126.76 ± 122.11	268.48 ± 162.65	<0.001^†^
Area (mm^2^)	13.23 ± 4.72	22.77 ± 4.72	0.001^†^
MLC density (cells/mm^2^)	8.71 ± 6.60	12.17 ± 8.06	0.01^†^

MLC, macrophage-like cell; BRVO, branch retinal vein occlusion; ONH, optic nerve head.

^†^Compared using Wilcoxon signed-rank test.

**Figure 4:**
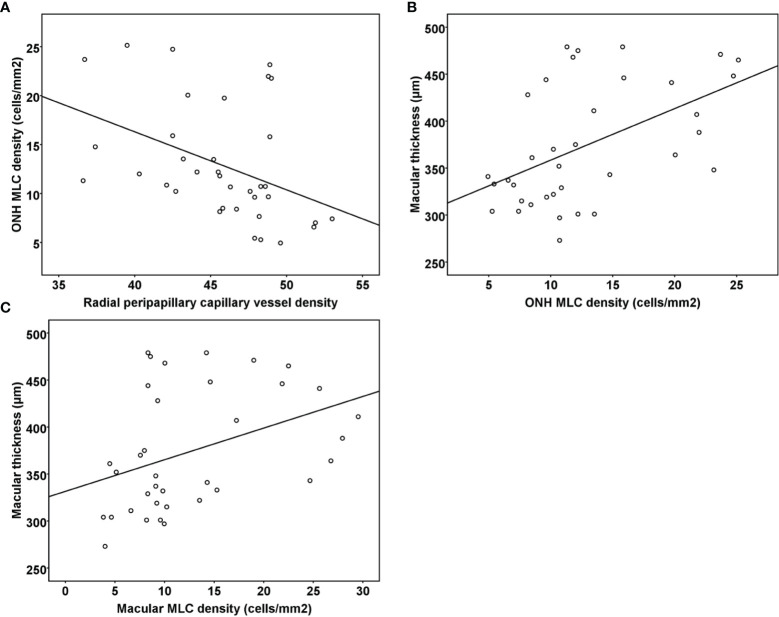
**(A)** Scatter plots demonstrating the association between radial peripapillary vessel density and ONH MLC density in the RVO eyes. **(B)** Scatter plots demonstrating the association between ONH MLC density and macular thickness in the RVO eyes. **(C)** Scatter plots demonstrating the association between macular MLC density and macular thickness in the RVO eyes. ONH, optic nerve head; MLC, macrophage-like cell; RVO, retinal vein occlusion.

Pearson ‘s correlation analysis showed that the ONH MLC density was negatively associated with the radial peripapillary capillary vessel density in all RVO eyes (r = −0.413, *p* = 0.012). However, no significant associations were found between the macular MLC density and macular vessel density in both the SCP and DCP (r = 0.059, *p* = 0.734; r = 0.253, *p* = 0.136, respectively). Both ONH and macular MLC densities were positively correlated with macular thickness in RVO (r = 0.505, *p* = 0.002; r = 0.385, *p* = 0.02, **Figure 4**). The MLC density was not associated with radial peripapillary vessel density, macular vessel densities, and macular thickness in the fellow eyes (all *p* > 0.05).

## Discussion

To the best of our knowledge, this is the first study investigating the distribution and clinical significance of MLCs using clinical en face OCT in acute RVO. The current study found that generalized increases in MLC density as well as morphologic changes were observed in eyes with acute RVO. More MLCs tended to aggregate in the perfused region rather than the non-perfused region. In addition, RVO patients with a higher density of MLCs tended to suffer from the thicker macula.

The morphologic changes and increased density of MLCs may indicate their activation and recruitment in RVO. A previous study also found that MLCs were larger and plumper in proliferative diabetic retinopathy, open-angle glaucoma, and CRVO, which was consistent with our study. The experimental animal model showed that the occlusion of the retinal vein caused ischemia and breakdown of the blood–retina barrier, followed by activation of microglia and aggregation of circulating macrophages (3). Conversely, depletion of microglia and macrophage in experimental RVO effectively reduced the expression of inflammatory cytokines in the ischemic retina (19). In terms of hyalocytes, the number of hyalocytes in the vitreous is low in physiological conditions (8). Conversely, cellular proliferation is promoted by cytokines like platelet-derived growth factor-BB under pathologic conditions (20). *In vivo* study showed that hyalocytes and glial cells were the predominant cell type in the internal limiting membrane of patients with idiopathic macular holes (21, 22). Changes in morphology and increase in density of hyalocytes were found after retinal damage (4). Although we could not differentiate microglia/hyalocytes based on the clinical en face OCT slab, cumulative evidence has shown that both cells play important roles in ocular inflammation. The activation and aggregation of MLCs may be generalized, even in the occlusion-unaffected region of BRVO. Our previous study and dozens of other studies have reported increased expression of inflammatory cytokines in the intraocular fluid of RVO patients (23, 24). Such findings suggested that the inflammation generated by a certain region could spread to the adjacent tissue or even the entire retina.

Another interesting finding was that fewer MLCs were observed in the non-perfused region compared to the perfused region in RVO. A previous study reported a similar finding that the MLCs were less likely to be found in the non-perfused areas in proliferative diabetic retinopathy (18). The human retina is a relatively immune-privileged organ because of the presence of the blood–retinal barrier and absence of the lymphatic system, local activation of immune-competent resident cells, and recruitments of systemic macrophages occur in response to retinal injury (25, 26). Thus, part of the MLCs could be monocyte-derived macrophage from the leaky vessel. However, when the vasculature is obstructed, it is difficult to deliver MLCs. On the other hand, a previous study suggested that MLCs preferred to avoid the ischemic region because their activation may be primarily reliant upon the glycolysis of the endothelial cells (18, 27).

The inverse correlation between the ONH MLC density and radial peripapillary vessel density indicates that the extent of capillary dropout of the tested retina may reveal the degree of MLC activation and aggregation. Interestingly, the macular MLC density did not correlate with macular vessel density in both the SCP and DCP. Segmentation error and the natural distribution of MLCs may result in the above phenomenon. Macular edema or exudation had a great influence on the segmentation of retinal superficial and deep vessel slabs. To maximally reduce the influence of segmentation error, we adjusted the boundary manually to their default position. However, the segmentation error may still exist and affect the accuracy of vessel density calculation. Moreover, we observed that MLCs on the inner limiting membrane were absent or sparse in the central macula of the normal eyes. Previous studies also found that the MLCs were sparse in the central macula and their density increased eccentrically in healthy eyes (10, 28). The boundaries of different retinal layers in the ONH region were clearer and thus ensure the accuracy of quantifying vessel density. The ONH region may be more reliable and accurate in revealing the association between MLCs and vessel density.

Macular edema is a frequent and vision-threatening manifestation secondary to RVO. We found that RVO patients with increased MLC density tended to be associated with the thicker macula. Previous studies reported that the intraretinal hyperreflective foci, which was thought to be an activated form of microglia or macrophage, was associated with increased retinal thickness and intraretinal fluid (29, 30). However, investigating the MLCs above the inner limiting membrane maximally avoids the influence of lipid exudation or retinal pigment epithelial cells. As we described above, MLCs may participate in the inflammatory process in ischemic conditions. Studies also found that microglia could be activated and migrate to alter the function of the blood–retinal barrier, releasing several pro-inflammatory cytokines while reducing the production of anti-inflammatory mediators at the proximity of the retinal vasculature (25, 31–33). Besides, previous studies had shown that the severity of macular edema was significantly associated with the aqueous and vitreous concentration of inflammatory cytokines in RVO (34). In terms of hyalocytes, a previous study found that they may be involved in various vitreoretinal diseases by increasing the production of VEGF under pathological conditions (35). VEGF is an essential contributing factor to macular edema, and eliminating its effect by anti-VEGF agent leads to successful resolution of the edema (36). Thus, hyalocytes may also contribute to the formation of macular edema in RVO. The correlation between MLC density and macular thickness indicated that activation and aggregation of MCLs might be one of the contributing factors to macular edema. *In vivo* study concerning the association between MLCs and macular edema may provide a better understanding of macular edema and develop subsequent innovative therapeutic strategies. Nevertheless, further investigation is needed to verify the association between MLCs and macular edema.

There are several potential limitations to our study. The activation of microglia and macrophage was characterized by a change in the morphology (4, 9). Although the morphology of MLCs in the RVO eye appeared less slender and plumper, we could not quantify the changes of MLC morphology using metrics like the size. A similar limitation was found in a previous study (18). Moreover, only 2 small regions were measured and used to quantify MLCs because of the limitation of the test field provided by the current OCTA device. This limitation could be improved with the advancement of OCTA technology. We did not correct the scale of OCT and OCTA images for individual differences in retinal magnification. However, we excluded subjects with refractive error >3 diopters [D] to limit such influence, and the symmetricity of axial length also minimizes such influence. Last but not least, the exact origin and function of MLCs in RVO remain unclear. Thus, further studies are needed to verify the roles of MLCs characterized by en face OCT.

In conclusion, activation and aggregation of MLCs visualized by clinical en face OCT were found on the inner limiting membrane of RVO eyes. The activation of MLCs might be generalized throughout the retina, although they tended to aggregate in the perfused region rather than the non-perfused region. The correlation between MLC density and macular thickness revealed that treatments targeting MLC activation or aggregation might potentially improve the neuroinflammation events detected in retinal degenerations.

## Data Availability Statement

The raw data supporting the conclusions of this article will be made available by the authors, without undue reservation.

## Ethics Statement

The studies involving human participants were reviewed and approved by the Institutional Review Board of the Zhongshan Ophthalmic Center. The patients/participants provided their written informed consent to participate in this study.

## Author Contributions

YKZ, XZ, and FW: conception and design of the study. YKZ, XZ, LM, YG, YS, ML, RY, and YNZ: data collection, analysis, and/or interpretation of the data. YKZ and XZ: drafting the article. All authors: revising it critically for important intellectual content. YKZ and FW: final approval of the version to be published. All authors read and approved the final manuscript.

## Funding

This work was supported by the National Natural Science Foundation of China [Grant Number: 82070970].

## Conflict of Interest

The authors declare that the research was conducted in the absence of any commercial or financial relationships that could be construed as a potential conflict of interest.

## Publisher’s Note

All claims expressed in this article are solely those of the authors and do not necessarily represent those of their affiliated organizations, or those of the publisher, the editors and the reviewers. Any product that may be evaluated in this article, or claim that may be made by its manufacturer, is not guaranteed or endorsed by the publisher.
